# P2Y2 Receptor Mediated Neuronal Regeneration and Angiogenesis to Affect Functional Recovery in Rats with Spinal Cord Injury

**DOI:** 10.1155/2022/2191011

**Published:** 2022-02-02

**Authors:** Ruidong Cheng, Genying Zhu, Chengtao Ni, Rui Wang, Peng Sun, Liang Tian, Li Zhang, Jie Zhang, Xiangming Ye, Benyan Luo

**Affiliations:** ^1^Department of Neurology & Brain Medical Center, The First Affiliated Hospital, Zhejiang University School of Medicine, Hangzhou, Zhejiang, China; ^2^Rehabilitation Medicine Center, Department of Rehabilitation Medicine, Zhejiang Provincial People's Hospital, Affiliated People's Hospital of Hangzhou Medical College, Hangzhou, Zhejiang, China; ^3^Graduate School, Bengbu Medical College, Bengbu, Anhui, China; ^4^Collaborative Innovation Center for Brain Science, Zhejiang University School of Medicine, Hangzhou, Zhejiang, China

## Abstract

The aim of this study was to investigate the effect of the P2Y2 receptor (P2Y2R) signaling pathway on neuronal regeneration and angiogenesis during spinal cord injury (SCI). The rats were randomly divided into 3 groups, including the sham+dimethyl sulfoxide (DMSO), SCI+DMSO, and SCI+P2Y2R groups. The SCI animal models were constructed. A locomotor rating scale was used for behavioral assessments. The apoptosis of spinal cord tissues was detected by TUNEL staining. The expression levels of P2Y2R, GFAP, nestin, Tuj1, and CD34 were detected by immunofluorescence staining, and the expression levels of TNF-*α*, IL-1*β*, and IL-6 were detected by enzyme-linked immunosorbent assay. The locomotor score in the model group was significantly lower than the sham group. The expression of P2Y2R was increased after SCI. The expression levels of TNF-*α*, IL-1*β*, and IL-6 were increased remarkably in the SCI model group compared with the sham group. The P2Y2R inhibitor relieved neuronal inflammation after SCI. Compared with the sham group, the apoptotic rate of spinal cord tissue cells in the model group was significantly increased. The P2Y2R inhibitor reduced the apoptosis of the spinal cord tissue. The expressions of CD34, Tuj1, and nestin in the model group were decreased, while the expressions of GFAP and P2Y2R were increased. The P2Y2R inhibitor reversed their expression levels. The P2Y2R inhibitor could alleviate SCI by relieving the neuronal inflammation, inhibiting the spinal cord tissue apoptosis, and promoting neuronal differentiation and vascular proliferation after SCI. P2Y2R may serve as a target for the treatment of SCI.

## 1. Introduction

Spinal cord injury (SCI) is a devastating neurological state causing major sensory motor and autonomic dysfunctions [1]. SCI leads to a series of molecular and cellular events, such as demyelination of surviving axons, free radical production, and release of nucleotides and excitatory amino acids, followed by neural tissue loss because of necrosis, apoptosis, macrophage infiltration, and inflammation [2]. Presently, the available treatments for SCI are limited, and only the supportive relief was provided for patients with lifetime disability [3]. Therefore, it is necessary to explore a feasible intervention approach for the clinical treatment of SCI.

A study has reported that after SCI, endogenous neural stem cells (ENSCs) are activated, generating progeny cells. However, these cells fail to effectively differentiate into functional neurons but astrocytes, which may be due to their impaired function after SCI [4]. The astrocytes participate in neural development in the central nervous system (CNS) [5]. During injury to the CNS, the astrocytes could result in reactive astrogliosis, characterized by the upregulated glial fibrillary acidic protein, and the proliferation of astrocytes at the lesion site, finally leading to the formation of glial scar [6]. The formation of a glial scar was one of important factors affecting the regeneration of neuron and nerve fiber [7, 8]. Recent studies have demonstrated that the astrocytes could be induced to differentiate into functional neurons by the intervention of NeuroD, Ascl1, or other signaling pathways [7, 8]. After SCI, the microcirculation and the microenvironment of the injury site play a crucial role in cell regeneration and repair [9], which determines the activation degree of astrocytes. The differentiation of astrocytes plays a key role in glial scar formation, spinal cord and axon repair, neuronal regeneration, and functional recovery of CNS. Therefore, how to induce ENSCs and astrocytes to differentiate into functional neurons is the key to improve the function of CNS after SCI.

Purines and their receptors, as an important neural signaling molecular, have participated in the transmission of peripheral and central nervous information and regulated the physiological activities of nerve tissue cells [9, 10]. Purinergic receptors included P1 adenosine receptors and P2 adenine nucleotide receptors, and the P2 receptors are divided into P2X (ionotropic) and P2Y (metabotropic) receptors [11]. It has been reported that astrocytes express several P2 receptor subtypes, including P2Y2 receptor (P2Y2R) [12]. Studies in human 1321 N1 astrocytoma cells expressing a recombinant P2Y2R have suggested that this receptor plays an important role in survival and neuroprotective mechanisms under pathological conditions [13]. Rodríguez-Zayas et al. [14] have demonstrated that the P2Y2R expression is increased in rats with SCI. Importantly, the expression and activation of P2Y2R are involved in the process of astrogliosis after CNS trauma [15, 16]. Nevertheless, there is no report on whether P2Y2R mediates angiogenesis, nerve repair, and regeneration after SCI.

In this study, we intended to investigate the effect of P2Y2R on the apoptosis of spinal cord tissue cells, neuron differentiation, angiogenesis, and neuronal proliferation. Thus, the SCI animal models were constructed followed by a series of experiments. Our findings may provide a new strategy for clinical treatment of SCI.

## 2. Materials and Methods

### 2.1. Animals and Grouping

Male Sprague-Dawley rats (260-300 g) were used in this study, which were housed at 21-25°C with a 12 h light/dark cycle, and were provided water and food ad libitum. All experiments were approved by the Institutional Animal Care and Use Committee of Zhejiang Provincial People's Hospital. The rats were randomly divided into 3 groups, including the sham+dimethyl sulfoxide (DMSO), SCI+DMSO, and SCI+P2Y2R groups, with 10 rats per group.

### 2.2. SCI Model Establishment

The SD rats were anesthetized by intraperitoneal injection of pentobarbital (30 mg/kg). The backs of the rats were shaved for routine disinfection. Following that, a midline skin incision of approximately 5 cm was made, and the muscles were separated to expose the vertebra. A miniature bone rongeur was used to resect the T9-T11 spinous processes and corresponding lamina. The spinal canal was opened at the T9-T11 level to fully expose the dorsal and bilateral sides of the spinal cord. The above procedures were performed with caution to ensure the dura mater remained intact. The spinal cord injury in the T9-T11 segment of rats was induced by the modified Mascis percussion device. In order to make the spinal cord of the three segments of T9-T11 bear a balanced impact force, a metal spacer with a width of 3 mm and a length and radian consistent with that of the spinal cord of the segments of T8-T10 was placed on the exposed dura of T9-T11 before the impact, and then, a weight of 10 g was dropped onto the metal spacer from a height. The weight and spacer were removed immediately after the blow, the wound was sutured layer by layer, and the incision was disinfected. The markers of the successful establishment of the model were as follows: visible seizure-like wagging of tails, retraction and fluttering of the hind limbs and body, and flaccid paralyses of the hind limbs.

For the sham group, only the spinous process and lamina were resected intraoperatively, and spinal cord shock injury was not performed. For the P2Y2R intervention group, after the model was established, the P2Y2R inhibitor (ar-c126313; 600 pmol/*μ*L; BOC Science, USA) was intrathecally injected at 10 *μ*L/d daily for 4 weeks.

### 2.3. Behavioral Assessments

Four observation time points were set, which were before injury, 1 day, 2 weeks, and 4 weeks after SCI. The rats were placed on an open test platform, and the locomotor activity of their hind limbs was observed continuously for 4 min by using the BBB (Basso, Beattie, and Bresnahan) locomotor rating scale at the 4 observation time points above. The scale is graded into 21 points with 0 indicating complete paralysis and 21 representing normal locomotion.

### 2.4. Hematoxylin and Eosin (HE) Staining

The spinal cord tissues were fixed in formaldehyde for 48 h and then rehydrated with different concentrations of ethanol, followed by embedding in paraffin. Then, the tissues were sectioned into 5 *μ*m slices, immersed in hematoxylin for 1 min, and then stained with eosin for 30 s. After being washed with water, the sections were dehydrated with graded alcohol, cleared in xylene, and then sealed for microscopic observation. After completion of the experiment, the rats were perfused with 4% paraformaldehyde. The spinal cord was retrieved from the rats and postfixed in 4% paraformaldehyde solution for another 4 h; and then, it was immersed in 30% sucrose solution overnight. The fixed spinal cord was sliced into 5 *μ*m slices, which were further stained by HE staining.

### 2.5. Terminal Deoxynucleotidyl Transferase-Mediated Nick End Labeling (TUNEL) Staining

The apoptosis of cells in spinal cord tissues was detected by TUNEL staining, according to the manufacturer's recommended instructions of TUNEL Color Labeling Apoptosis Detection Kit (11684817910; Roche Applied Science, USA). Spinal cord tissues were fixed in formaldehyde for 48 h and then rehydrated with different concentrations of ethanol, followed by embedding in paraffin. Then, the paraffin block was cut into 4-7 *μ*m thick sections and then deparaffinized with xylene (2 × 15 minutes) and rehydrated with graded concentrations of ethanol. After that, the sections were performed with antigen retrieval in boiling sodium citrate (0.01 M) in a pressure cooker and then treated with the TUNEL reaction mixture (50 *μ*L TdT + 450 *μ*L fluorescein-labeled dUTP) at 37°C for 1 h in a dark wet box. The sections were stained with DAB, and the numbers of TUNEL-positive cells were assessed using a fluorescence microscope. The apoptotic index was calculated as the percentage of TUNEL-positive cells in a section of 5 randomly selected areas of the specimens.

### 2.6. Immunofluorescence Staining

For immunofluorescence staining, spinal cord tissues were fixed in formaldehyde for 48 h and then rehydrated with different concentrations of ethanol, followed by embedding in paraffin. Spinal cord sections (4-7 *μ*m thick) from each specimen were deparaffinized with xylene (2 × 15 minutes) and then incubated in graded concentrations of ethanol (100%, 95%, 85%, and 75%). The sections were then subjected to antigen retrieval in boiling sodium citrate (0.01 M) in a pressure cooker, followed by phosphate buffer solution (PBS) washing. After that, the samples were incubated with the primary antibodies overnight at 4°C. The primary antibodies included anti-P2Y2R (1 : 500; P6612; Sigma, USA), glial fibrillary acidic protein (GFAP; 1 : 500; PAA068Ra01; USCN Life Science; Wuhan, China), nestin (1 : 500; PAA500Ra01; USCN Life Science; Wuhan, China), Tuj1 (1 : 1000; ab68193; Abcam, USA), and CD34 (1 : 1000; ab81289; Abcam, USA). The next day, the sections were washed with PBS and then incubated with the fluorescent-labeled secondary antibodies for 30 min at 37°C. After 3 rinses with PBS, DAPI staining was performed, and the nuclei were counterstained with 1 g/mL Hoechst (BioSharp, China) for 5 minutes. The immunofluorescence imaging was conducted using a fluorescence microscope.

### 2.7. Enzyme-Linked Immunosorbent Assay (ELISA)

The expression levels of tumor necrosis factor-*α* (TNF-*α*), IL-1*β,* and IL-6 were detected by ELISA. The spinal cord samples were homogenized and centrifuged at 1000*g* for 20 min. The liquid supernatant was collected and tested at a wavelength of 450 nm according to the manufacturer's instructions of ELISA kits (R&D Systems, Minneapolis, Minn., USA).

### 2.8. Western Blotting

The spinal cord tissues were lysed by homogenization in 300 *μ*L of lysis buffer. Proteins were separated on 12% sodium dodecyl sulfate polyacrylamide gel electrophoresis (SDS-PAGE) gels. Then, the proteins in the gels were transferred to polyvinylidene difluoride (PDVF) membranes. The membranes were blocked for 2 h, followed by incubation with monoclonal antibodies against GAPDH (1 : 3000) and P2Y2R (1 : 200) at 4°C overnight. After 3 washes with TBST, horseradish peroxidase-conjugated goat anti-rabbit IgG was added for an additional 1.5 h. Then, the blots were visualized using an enhanced chemiluminescence (ECL) system.

### 2.9. Biotinylated Dextran Amine (BDA) Tracing

Two weeks after the model was constructed, the rats were fixed on the stereotaxic apparatus after anesthesia, and the scalp in the parietal area was cut lengthwise. The periosteum was cut and pushed around. Then, the skull was swabbed with hydrogen peroxide. In reference to the location map of the rat brain in *vivo*, the anterior fontanelle was taken as the origin, and 8 locations were selected for drilling. Then, 1 *μ*L of 5% BDA solution was injected into the motor cortex of rats with a microinjector, and the depth of injection was about 3.5 mm from the surface of the skull. The injection time was set for 5 min. After the injection, the scalp was sutured. After 2 weeks, the spinal cord tissue samples below the injured segment were collected for BDA fluorescence staining to observe the nerve fibers of the corticospinal tract.

### 2.10. Statistical Analysis

Results were analyzed by ANOVA test using GraphPad Prism 6 (Graph Pad Software Inc., San Diego, CA, USA). Data were expressed as means ± standard deviations (SD). Differences were considered to be significant at *p* < 0.05.

## 3. Results

### 3.1. The Expression Level of P2Y2R Was Increased after SCI

**Figure 1:**
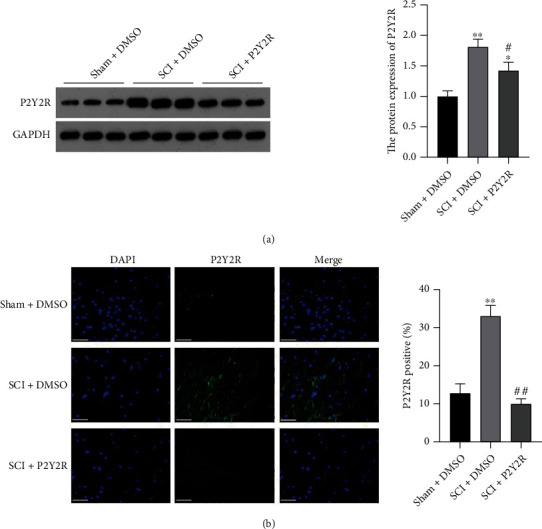
(a) The expression level of P2Y2R in spinal cord tissue detected by western blot. (b) The expression level of P2Y2R in spinal cord tissue detected by immunofluorescence staining. Scale bar = 50 *μ*m. ^∗^*p* < 0.05 and ^∗∗^*p* < 0.01 compared with the sham+DMSO group; ^#^*p* < 0.05 and ^##^*p* < 0.01 compared with the SCI+DMSO group.

The expression level of P2Y2R in spinal cord tissues in three groups was detected by western blot and immunofluorescence staining. As shown in Figures 1(a) and 1(b), the expression level of this receptor was increased obviously in the model group in comparison with the sham group. When the P2Y2R inhibitor was added, its expression was significantly decreased compared with the model group.

### 3.2. BBB Locomotor Score

**Figure 2:**
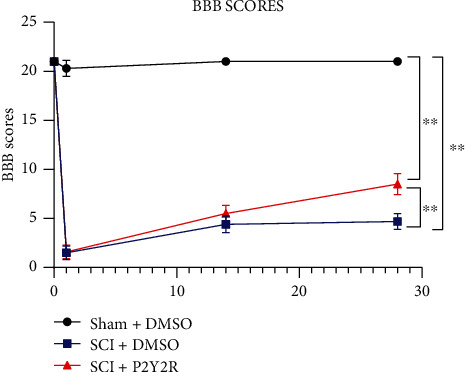
Recovery of motor function assessment. The recovery of the motor function was assessed with the BBB scoring method. After 2 weeks and 4 weeks of modeling, ^∗∗^*p* < 0.01 compared with the sham+DMSO group; ^#^*p* < 0.05 compared with the SCI+DMSO group.

On the first day of modeling, rats in the model group and the P2Y2R inhibitor group almost lost their hind limb motor ability compared with the sham group. After 2 weeks and 4 weeks of modeling, the hind limb motor ability of rats in the model group and P2Y2R inhibitor group recovered somewhat, and the recovery effect in the P2Y2R inhibitor group was better than that in the model group (Figure 2).

### 3.3. P2Y2R Inhibitor Reduced Cell Apoptosis in Spinal Cord Tissue

**Figure 3:**
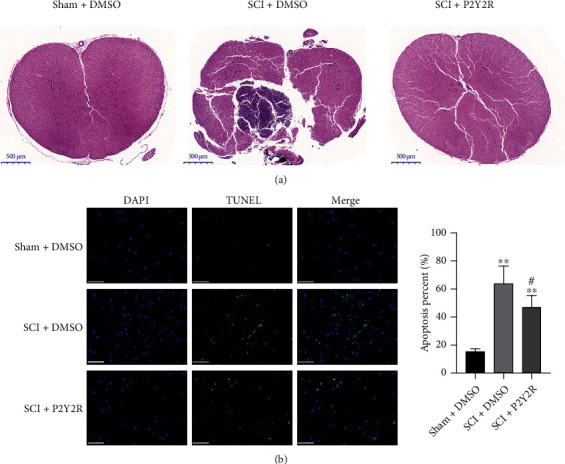
(a) The results of HE staining. (b) The apoptosis of spinal cord tissue detected by TUNEL assay. Scale bar = 50 *μ*m.^∗∗^*p* < 0.01 compared with the sham+DMSO group; ^#^*p* < 0.05 compared with the SCI+DMSO group.

HE staining of the spinal cord showed that the gray matter of the spinal cord in the sham+DMSO group was clearly demarcated, and the nerve cells in the gray matter were large, with abundant Nissl bodies and nucleolus. In the SCI+DMSO group, the damaged area was collapsed, and some nerve cells and fibers disappeared and were replaced by glial cells, resulting in patches of glial scar. The damage degree of the SCI+P2Y2R group was lighter than the SCI+DMSO group (Figure 3(a)).

The results of TUNEL assay in spinal cord tissue of rats in each group are shown in Figure 3(b). Compared with the sham group, the apoptotic rate of spinal cord tissue cells in the model group was significantly increased. Compared with the model group, the degree of spinal cord cell apoptosis was significantly decreased in the P2Y2R inhibitor group. The results indicate that the P2Y2R inhibitor could reduce the apoptosis of the spinal cord tissue, thus effectively alleviating the SCI.

### 3.4. P2Y2R Inhibitor Relieved Neuronal Inflammation after SCI

**Figure 4:**
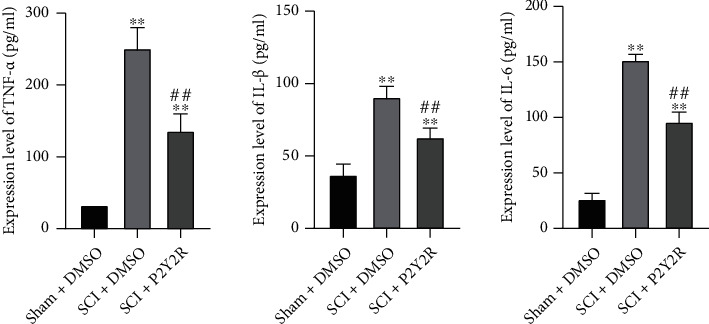
The expression levels of TNF-*α*, IL-1*β,* and IL-6 detected by ELISA. ^∗^*p* < 0.05 and ^∗∗^*p* < 0.01 compared with the sham+DMSO group; ^##^*p* < 0.01 compared with the SCI+DMSO group.

The proinflammatory cytokines, including TNF-*α*, IL-1*β*, and IL-6, had increased remarkably in the SCI model group compared with the sham group. Interestingly, P2Y2R inhibitor treatment could significantly reduce the release of TNF-*α*, IL-1*β*, and IL-6 when compared with the model group (Figure 4). This result indicated that the P2Y2R inhibitor could relieve neuronal inflammation of SCI.

### 3.5. P2Y2R Inhibitor Inhibited Glial Scar Formation and Promoted Nerve Fiber Growth after SCI

**Figure 5:**
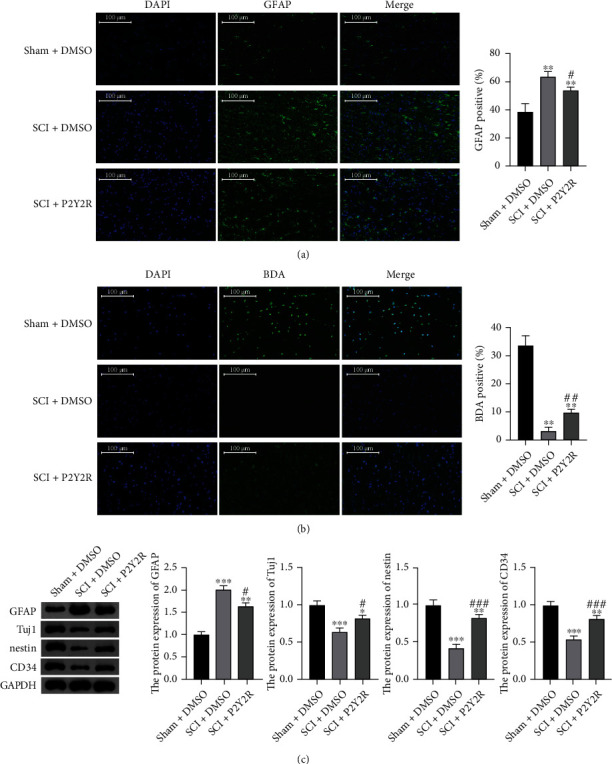
(a) The expression levels of GFAP detected by immunofluorescence staining. (b) Results of BDA staining. Scale bar = 100 *μ*m. (c) The relative protein expression of GFAP, Tuj1, nestin, and CD34 measured by western blotting. ^∗∗^*p* < 0.01 compared with sham+DMSO group; ^#^*p* < 0.05 and ^##^*p* < 0.01 compared with the SCI+DMSO group.

The expression level of GFAP was detected through immunofluorescence staining and western blot, as shown in Figures 5(a) and 5(c). In comparison with the sham group, the expression of GFAP was significantly increased. Nevertheless, after P2Y2R inhibitor treatment, the expression of GFAP was decreased in the spinal cord of rats when compared with the model group. These results suggested that the P2Y2R inhibitor may inhibit glial scar formation after SCI.

The results of BDA staining of the spinal cord tissue are shown in Figure 5(b). As can be seen from the figure, compared with the sham group, the expression of BDA in the spinal cord of rats in the model group was significantly decreased. When compared with the model group, the expression of BDA was significantly increased in the spinal cord of rats treated with P2Y2R inhibitor. The results indicated that the P2Y2R inhibitor could promote nerve fiber proliferation after SCI to alleviate SCI.

### 3.6. P2Y2R Inhibitor Promoted Neuronal Differentiation and Angiogenesis after SCI

**Figure 6:**
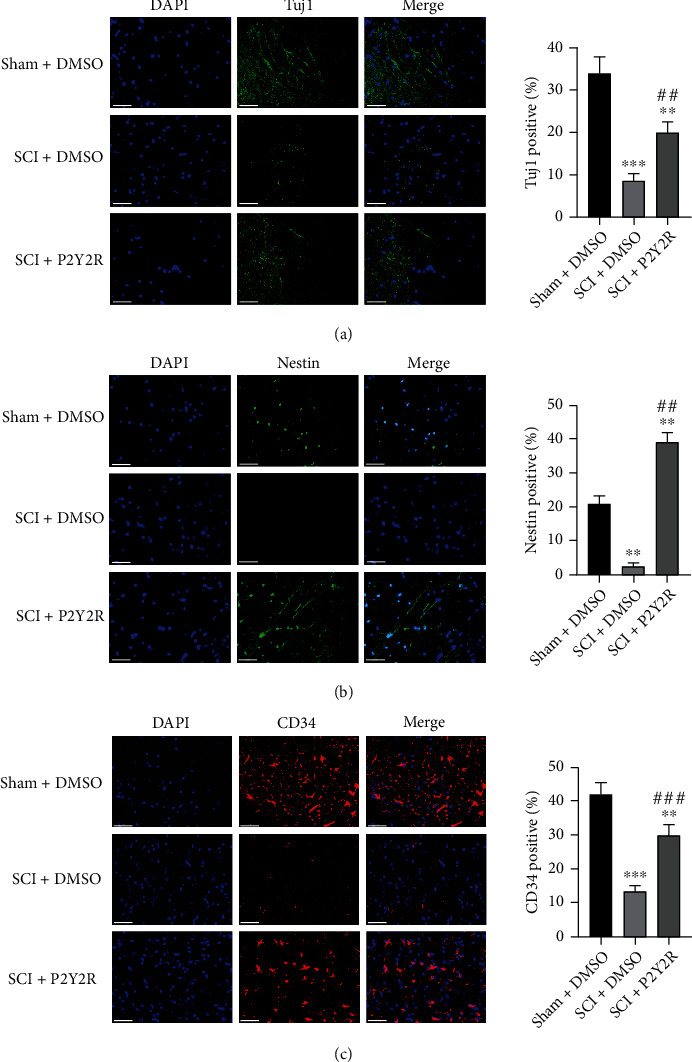
The expression levels of Tuj1, nestin, and CD34 detected by immunofluorescence staining. Scale bar = 50 *μ*m. ^∗^*p* < 0.05 and ^∗∗^*p* < 0.01 compared with the sham+DMSO group; ^#^*p* < 0.05 and ^##^*p* < 0.01 compared with the SCI+DMSO group.

The expression levels of Tuj1, nestin, and CD34 were detected through immunofluorescence staining and western blot, as shown in Figures 6 and 5(c). In comparison with the sham group, the expressions of Tuj1, nestin, and CD34 in the spinal cord of rats in the model group were significantly decreased. Nevertheless, after P2Y2R inhibitor treatment, the expressions of Tuj1, nestin, and CD34 were significantly increased in the spinal cord of rats when compared with the model group. These results suggested that the P2Y2R inhibitor may promote neuronal differentiation and angiogenesis after SCI, thereby alleviating spinal cord injury effectively.

## 4. Discussion

SCI can lead to sensory impairment and paraplegia. Recently, the studies on pathological mechanisms have explored many new therapeutic methods for SCI. However, there is still a need to find more precise and specific treatment targets for these adverse outcomes [17]. In this study, our results showed that the expression of P2Y2R was increased after SCI in rats. The P2Y2R inhibitor could relieve the neuronal inflammation of SCI, reduce the apoptosis of the spinal cord tissue, and promote neuronal differentiation and vascular proliferation and neuronal proliferation after SCI.

The presence of P2Y1, P2Y2, P2Y4, P2Y6, P2Y12, and P2X2 receptors in the adult spinal cord was evident. P2Y receptors have been associated with survival responses in nervous tissues [18]. This family of receptors is considered the strong modulator of normal and pathological processes in the CNS [19]. Suramin was originally found to inhibit trypanocidal activity [20]. It has been shown to be a potent inhibitor of various hydrolytic and oxidative enzymes by interfering with the binding of ATP. P2XRs are activated by ATP; thus, suramin was later identified as a P2XR antagonist [21]. Suramin has been reported to inhibit P2YRs except for P2Y4 and P2Y6Rs which are insensitive [22]. In this study, after 2 weeks and 4 weeks of modeling, the hind limb motor ability of rats in the P2Y2R inhibitor (suramin) group recovered somewhat. However, in a previous study, the intrathecal administration of antagonists (suramin and PPDS) and the blockade of the P2 receptor activation do not improve locomotor behavior in the SCI rats. The decrease in reactive gliosis and the increases in the lesion cavity, caused by the infusion of antagonists, may be associated with the lack of locomotor improvement [19].

On the first day of modeling, rats in the model group and the P2Y2R inhibitor group almost lost their hind limb motor ability compared with the sham group. After 2 weeks and 4 weeks of modeling, the hind limb motor ability of rats in the model group and P2Y2R inhibitor group recovered somewhat, and the recovery effect in the P2Y2R inhibitor group was better than that in the model group.

Under various physiological and pathological conditions, extracellular nucleotides are released from cells in the CNS [23]. These nucleotides could activate P2 receptors on the surface of adjacent cells [20]. Previous studies have reported that metabotropic P2Y2R was upregulated in different cellular and animal models of injury [14, 21], suggesting that this receptor plays a critical role in the cellular response to tissue damage [24]. In accordance with the report above, our results also revealed that the expression of P2Y2R was elevated in the SCI model group, further supporting the ideas above.

CD34 is one of the hematopoietic markers, which could regulate migration and trafficking of hematopoietic progenitor cells [25]. CD34 may eventually migrate to the CNS and differentiate into microglia [26]. After CNS injury, CD34-expressing microglia could be found in affected regions, characterized by the damage of microgliosis and blood-brain barrier [27]. In this study, CD34 was found to be downregulated in SCI model rats, while was upregulated in the P2Y2R inhibitor group. This result suggested that the P2Y2R inhibitor may improve angiogenesis after SCI, evidenced by increased CD34 expression.

Tuj1 and nestin are neuron-specific molecular markers, which play important roles during the neural cell maturation. Tuj1 is a beta III microtubule specifically associated with neural differentiation, whose expression is elevated during the early stages of neural differentiation [28]. Nestin is an intermediate filament type VI, and is mainly expressed by neural cells. It affects the radial growth of axons, as well as the survival, proliferation, and self-renewal of neural cells [29]. It has been found to be upregulated in the early stage of neural differentiation [30]. In this study, the downregulation of Tuj1 and nestin in the model group suggested the impaired neuronal differentiation after SCI. After P2Y2R inhibitor treatment, the expression of Tuj1 and nestin was increased, suggesting that suppression of P2Y2R may improve the neuronal differentiation after SCI.

GFAP is an essential component of the astrocyte cytoskeleton. It is one of the best biomarkers for the activation of astrocytes following injury or stress in the CNS [31]. It has been reported that the expression of GFAP negatively correlates with the formation of the inhibitory glial scar. The reduction of the GFAP expression is more desirable for the repair of SCI [32]. In this study, GFAP expression was reduced in the P2Y2R inhibitor group, suggesting the SCI repair function of the P2Y2R inhibitor.

Future studies should identify other molecular mechanisms that are involved in the P2Y2R signaling pathway in rats with SCI. Neural stem cells are a source of glial scar astrocytes with beneficial functions. Nowadays, SCI is often considered an attractive indication for the development of stem cell transplantation therapies. The discovery of ENSCs in the adult spinal cord offers hope for noninvasive treatment of SCI. Thus, whether the P2Y2R inhibitor has an effect on the ENSC activation needs further investigation [7].

Furthermore, P2Y2R knockout mice could be used to investigate the role of P2Y2R signaling in impair corticospinal tract function, increases calcium load, and the area of spinal injured areas. In light of the relationship between P2Y2R and neuronal regeneration, it will be interesting to explore the potential applications of P2Y2R inhibitors and other molecules that are unique to neuronal repair in spinal cord disease conditions.

## 5. Conclusion

**Figure 7:**
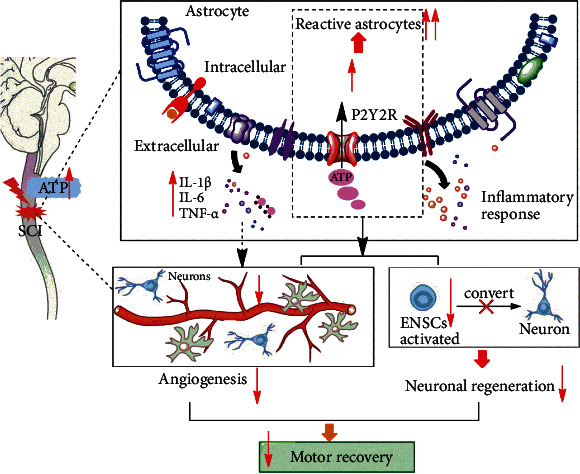
The P2Y2R signaling pathway could play a considerable role in the development of neuronal regeneration and angiogenesis to affect functional recovery in rats following SCI.

In summary, our study demonstrated again that P2Y2R was overexpressed after SCI in rats. The P2Y2R inhibitor could alleviate SCI by relieving the neuronal inflammation, inhibiting the spinal cord tissue apoptosis, and promoting neuronal differentiation and angiogenesis after SCI (Figure 7). The P2Y2R may serve as a target for the treatment of SCI.

## Data Availability

The data used to support the findings of this study are available from the corresponding authors upon request.
